# *miR-34a* screened by miRNA profiling negatively regulates *Wnt*/*β-catenin* signaling pathway in Aflatoxin B1 induced hepatotoxicity

**DOI:** 10.1038/srep16732

**Published:** 2015-11-16

**Authors:** Liye Zhu, Jing Gao, Kunlun Huang, Yunbo Luo, Boyang Zhang, Wentao Xu

**Affiliations:** 1 Laboratory of Food Safety, College of Food Science and Nutritional Engineering, China Agricultural University, Beijing, China, 100083; 2 Beijing Laboratory for Food Quality and Safety, Beijing, P. R. China

## Abstract

Aflatoxin-B1 (AFB1), a hepatocarcinogenic mycotoxin, was demonstrated to induce the high rate of hepatocellular carcinoma (HCC). MicroRNAs (miRNAs) participate in the regulation of several biological processes in HCC. However, the function of miRNAs in AFB1-induced HCC has received a little attention. Here, we applied Illumina deep sequencing technology for high-throughout profiling of microRNAs in HepG2 cells lines after treatment with AFB1. Analysis of the differential expression profile of miRNAs in two libraries, we identified 9 known miRNAs and 1 novel miRNA which exhibited abnormal expression. KEGG analysis indicated that predicted target genes of differentially expressed miRNAs are involved in cancer-related pathways. Down-regulated of *Drosha*, *DGCR8* and *Dicer 1* indicated an impairment of miRNA biogenesis in response to AFB1. *miR-34a* was up-regulated significantly, down-regulating the expression of *Wnt/β-catenin* signaling pathway by target gene *β-catenin*. *Anti-miR-34a* can significantly relieved the down-regulated *β-catenin* and its downstream genes, *c-myc* and *Cyclin D1*, and the S-phase arrest in cell cycle induced by AFB1 can also be relieved. These results suggested that AFB1 might down-regulate *Wnt/β-catenin* signaling pathway in HepG2 cells by up-regulating *miR-34a*, which may involve in the mechanism of liver tumorigenesis.

Aflatoxins were highly toxic, mutagenic, and carcinogenic compounds, which produced by fungi *Aspergillus flavus* and *Aspergillus parasiticus*^1^. The aflatoxins were discovered as a contaminant of human and animal food in the late 1950s and early 1960s^2^. Aflatoxins are potent liver toxins, lethal when consumed in large doses. Studies showed that dietary exposure to aflatoxins is one of the major risk factors for HCC, the most frequent liver cancer in the world^3^. More than 600,000 people die each year from HCC, mostly (48%) in developing countries^4^. AFB1, the major aflatoxin product, is a genotoxic hepatocarcinogenic compound which may also cause tumors in other organs, such as colon and kidney^5^. It is bio-activated in liver by cytochrome P450 and its epoxide metabolite attacks DNA, forming AFB1-DNA adducts^6^ that might evolve to secondary injuries such as apurinic sites (AP) or imidazole AFB1 formamidopyrimidine opened rings (AFB1-FAPY)^7^. Therefore, the removal or inactivation of this toxic fungal metabolite is a major concern to salvage-contaminated foodstuffs and feedstuffs^8^. The human hepatocellular carcinoma cell line HepG2 has been widely used as a model system to evaluate toxic effects of various substances, especially for the study of the most potent hepatocarcinogen, AFB1^9^,^10^. MiRNAs are endogenous, non-coding small RNAs that usually are 21–30 nt^11^. MiRNAs can regulate gene expression at post-transcriptional level by degrading target mRNA or inhibiting translation as a result of complementary matching between miRNAs and specific sites in target genes. A large fraction of protein-coding genes can be miRNA targets, while a single miRNA can target hundreds to a thousand or more mRNAs as well. In the recent years, several studies revealed that miRNAs aberrantly expressed in human HCC in comparison with matched non-neoplastic tissue^12^. Furthermore, results from some reports suggested that changes in the expression of miRNAs may occur early in a variety of essential cellular processes, including cell growth, differentiation, metabolism and apoptosis^13^, and these changes may be related to specific etiological factors, such as AFB1. Some studies showed that genotoxical environmental agents, including AFB1, caused a variety of non-genotoxic alterations. These still preliminary evidences raised the possibility of using miRNAs as early markers for aflatoxins exposure^14^.

Recently, *miR-34a* has been demonstrated to be a key direct transcriptional target of *p53* in most HCC^15^,^16^. Many of the biological targets of *miR-34a* have been recently identified. Studies showed that the ectopic expression of *miR-34a* induces cell-cycle arrest, senescence and apoptosis by regulation of critical cell cycle motors or apoptosis inhibitors including *CDK4/6*, *cyclin E2* (*CCNE2*), *cyclin D1*(*CCND1*), *E2F3*, and *Bcl-2*^17^,^18^. Interestingly, *miR-34a* also exerts tumor suppressor activity by regulating *Wnt/β-catenin* signaling which is a master regulator of cell proliferation, differentiation and movement^19^. Aberrant regulation of the *Wnt/β-catenin* signaling pathway by the mutation of one of the critical members of this pathway appears to play an important role in the development of hepatocellular cancers^20^. However, mechanisms of the regulation of miRNA in hepatocellular cancers development remain to be clarified.

Considering the effects of AFB1 as one of the most important reasons in HCC, we hypothesized that AFB1 might also trigger the differential expression of miRNAs which contribute to hepatocellular cancer development. Moreover, there are a lack of knowledge on the relationship between miRNAs and AFB1 *in vitro*, which is important to explain the role of miRNA in carcinogenesis. Based on these, by using Illumina deep sequencing, the changes of miRNAs profiling induced by AFB1 can be well studied. What’s more, the role of *miR-34a* will be explored in the hepatotoxicity induced by AFB1.

## Methods

### Cell culture and treatment

The human HCC cell lines HepG2 were cultured in monolayer in Dulbecco’s Modified Eagle’s Medium (DMEM, Neuronbc, Beijing) supplemented with 10% of fetal bovine serum (FCS, Hyclone, USA) and 1% of antibiotics (100 U/mL Penicillin Streptomycin Amphotericin B, Maichen). Cells were grown at 37 °C and 5% CO_2_ in a humidified atmosphere. For cell counting and subculture, the cells were dispersed with trypsin. HepG2 cells were treated with AFB1 at different concentrations of 0 and 10 μg/mL for 24 h. We labeled the 10 μg/mL treatment as group N (N1 and N2 for duplication), while the control as group CK (CK1 and CK2 for duplication). AFB1 were dissolved in DMSO and added to the culture media. The final concentration of DMSO in the media was less than 0.1%. Every group was designed two repeats, while the R^2^ were 0.971 and 0.964 of CK and the AFB1 treatment group, respectively.

### RNA extraction

About 5.0 × 10^6^ cells per sample were used for RNA isolation using miRcute miRNA Isolation Kit (Tiangen, Beijing) according to the manufacturer’s protocol. RNA degradation and contamination were monitored on 1% agarose gels. RNA purity was checked using the Nano Photometer® spectrophotometer (IMPLEN, CA, USA). RNA concentration was measured using Qubit® RNA Assay Kit in Qubit® 2.0 Flurometer (Life Technologies, CA, USA), while the RNA integrity was assessed using the RNA Nano 6000 Assay Kit of the Agilent Bioanalyzer 2100 system (Agilent Technologies, CA, USA) with the parameters: RIN ≥ 7.5, concentration ≥ 200 ng/μL.

### Library preparations for Small RNA sequencing

RNA samples were stored at −80 °C and sequenced with the Illumina HiSeq^TM^2000/MiSeq platform. An amount of 3 μg total RNA per sample was used as input material for the small RNA library. Sequencing libraries were generated using NEB Next Multiplex Small RNA Library Prep Set for Illumina (NEB, USA.) following manufacturer’s recommendations and index codes were added to attribute sequences to each sample. Briefly, NEB 3′ SR Adaptor was direct, and specifically ligated to 3′ end of miRNA, siRNA and piRNA. After the 3′ ligation reaction, the SR RT Primer hybridized to the excess of 3′ SR Adaptor (that remained free after the 3′ ligation reaction) and transformed the single-stranded DNA adaptor into a double-stranded DNA molecule. This step is important to prevent adaptor-dimer formation, besides, dsDNAs are not substrates for ligation mediated by T4 RNA Ligase 1 and therefore do not ligate to the 5′SR Adaptor in the subsequent ligation step. 5′ends adapter was ligated to 5′ends of miRNAs, siRNA and piRNA. Then first strand cDNA was synthesized using M-MuLV Reverse Transcriptase (RNase H^−^). PCR amplification was performed using Long Amp Taq 2×Master Mix, SR Primer for illumina and index (X) primer. PCR products were purified on a 8% polyacrylamide gel (100 V, 80 min). DNA fragments corresponding to 140 ~ 160 bp (the length of small noncoding RNA plus the 3′ and 5′ adaptors) were recovered and dissolved in 8 μL elution buffer. At last, library quality was assessed on the Agilent Bioanalyzer 2100 system using DNA High Sensitivity Chips.

The clustering of the index-coded samples was performed on a cBot Cluster Generation System using TruSeq SR Cluster Kit v3-cBot-HS (Illumia) according to the manufacturer’s instructions. After cluster generation, the library preparations were sequenced on an Illumina Hiseq 2500/2000 platform and 50 bp single-end reads were generated.

### Target gene predictions

Target predictions, determined with avariety of algorithms including DIANA-microT, miRanda, PicTar, and TargetScanS, were collected for the validated miRNA with the miRGendatabase (version 4.0). Multihit miRNA target analysis was also performed with miRanda alone (http://www.microRNA.org)to enrich for target genes with three or more target sites for the validated schizophrenia-associated miRNA. The frequency of miRNA targeting for any given target gene was determined for the pooled gene list of validated miRNA with PASW Statistics 18, (SPSS, Chicago, Illinois; IBM, Armonk, New York). In the multihit analysis, target genes with more putative miRNA binding sites were assumed to have more potential for post-transcriptional regulation and greater intensity than those with only one or two.

### GO and KEGG Enrichment Analysis

Pathway analysis of these lists was achieved with the functional annotation tools on the Database for Annotation, Visualization and Integrated Discovery (DAVID; http://david.abcc.ncifcrf.gov/)^21^,^22^. Gene Ontology (GO) enrichment analysis was used on the target gene candidates of differentially expressed miRNAs. GOseq based Wallenius non-central hyper-geometric distribution^23^, which could adjust for gene length bias, was implemented for GO enrichment analysis.

### Quantification of miRNAs and mRNAs

Semiquantitative real-time PCR (qRT-PCR) was performed on 200 ng of total RNA extracts that had been polyadenylated and reverse transcribed into cDNA using an anchored oligo (dT) primer (Tiangen, Beijing, China). The miRNA was transcribed into first-strand cDNA using miRcute miRNA first-strand cDNA synthesis kit (Tiangen, Beijing, China). The miRNA primers were listed in the supplementary Table 1A.

mRNA was transcribed into first-strand cDNA using Quantscript RT Kit (Tiangen, Beijing, China). qRT-PCR was run using the RealMasterMix (SYBR green I) (Tiangen, Beijing, China). The genes include *Drosha, DRCG8, Dicer1* and the target genes of some miRNAs. RT-PCRs were run on the CFX96 Real-time PCR machine (BIO-RAD, Richmond, CA). The primers were marked in the supplementary Table 1B. Each RT reaction contained 11.25 uL 2.5 × RealMasterMix, 0.3 uL stem loop RT specific primer, 200 ng sample in 25 uL according to the Kit Introduction. Target sequences were amplified by being incubated at 94 °C for 2 min, followed by 40 cycles of 94 °C for 20s and 58 °C for 34s. *β-actin* and *5S RNA* were used as endogenous normalization control for genes and miRNA separately. All assays were analyzed using the delta–delta–Ct method.

### Transfection of miR-34a inhibitor

*MiR-34a* inhibitor oligonucleotide (ACAACCAGCUAAGACACUGCCA) and negative control were chemically synthesized bygenepharma (Shanghai, China). HepG2 cells were transfected with 150 nM oligonucleotides using HTF of Nuolanxin (Beijing, China), according to the manufacturer’s protocol. Six hours after transfection, the cells were treated with 5, 10 and 15 μg/mL AFB1 untreated for an additional 24 h. The cells were harvested for subsequent experiments.

### Cell-cycle analysis

The cells treated by AFB1 after the miRNA inhibitor and NC transfections were harvested and washed once in phosphate-buffered saline, and fixed in 70% ethanol over night. The DNA content was then examined using the Cell Cycle Analysis Kit of Beyotime (Beijing, China) and analyzed using BD FACS Calibur FlowCytometer. The data were analyzed by ModiFit analysis software.

### Statistics

The data were expressed as the means ± standard deviation. The experiments were repeated at least twice, and each experiment included at least triplicate treatments. The data from different treatments were subjected to an analysis of variance (ANOVA), and the comparisons of the means were performed using Duncan’s multiple range test. All of the statistical analyses were performed using the SPSS 16.0 software program. Differences with values of *p* < *0.05* were considered to be significant.

## Results

### Summary of small RNA sequencing data

**Figure 1:**
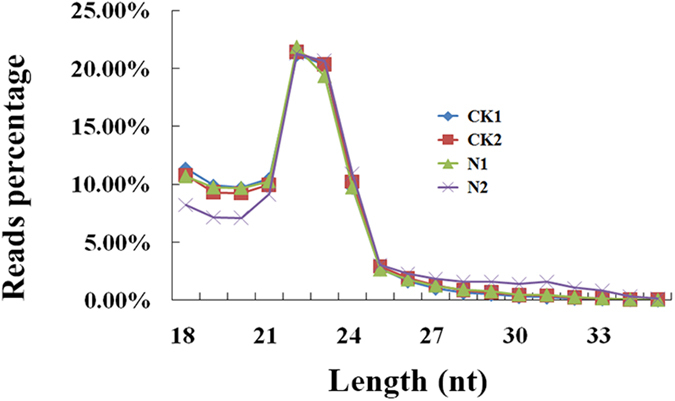
Small RNA reads percentage of different length distribution in CK group and N group. HepG 2 cells were treated with AFB1 (0 and 10 μg/ml), denoted as CK and N group, respectively for 24 hours.

Total RNA was extracted from each group and thus two datasets were obtained from CK and treatment (CK1, 6889737 reads; CK2, 6528991 reads; N1, 5652409 reads; N2, 9962058 reads), respectively. Clean reads (about 90% of total reads) were obtained by removing reads containing ploy-N, with 5′adapter contaminants, without 3′adapter or the insert tag, containing ploy A or T or G or C and low quality reads from raw data (Table 1). Then, we chose a certain range of length (18–35 nt) from clean reads to do all the downstream analyses. Analyses of small RNA length distribution peaked at the size of 22 nt as shown in Fig. 1. The small RNA tags were mapped to reference sequence by Bowtie^24^ without mismatch to analyze their expression and distribution on the reference. Mapped small RNA tags were used to looking for known miRNA. miRBase20.0 was used as reference, modified software mirdeep2^25^ and srna-tools-cli were used to obtain the potential miRNA and draw the secondary structures. Custom scripts were used to obtain the miRNA counts as well as base bias on the first position of identified miRNA with certain length and on each position of all identified miRNA respectively.

The matched reads were used to identify mature miRNAs, and the numbers of their reads were accounted. The reads that did not yield a match were used to predict novel miRNAs using MIREAP. The numbers of miRNA reads were normalized by Tags per million (TPM) values^26^ (TPM = (readCount *1,000,000)/libsize) to express miRNAs in CK and N comaprable in one table.

### Novel miRNA analyses

**Figure 2:**
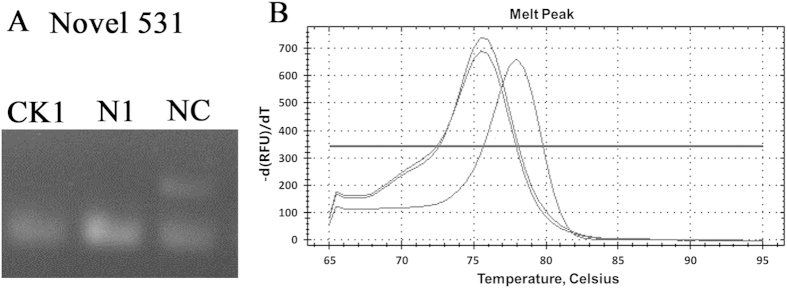
PCR analyses of the novel miRNA. (**A**) the novle miRNA of CK1, N1 and NC was detected by PCR. (**B**) The melt peak of the novle miRNA of qRT-PCR product in CK1, N1 and NC group was detected.

A number of criterions were used for evaluating whether a small RNA was a genuine miRNA, such as formation of a stable hairpin structure, lower minimal free energies for hairpin structure of its precursors, and detection of miRNAs^27^. Given these analyses, novel miRNAs were identified and examined by real-time PCR. The negative peak and the positive peak were different. Based on this, the novel miRNA was existing (Fig. 2, supplementary Fig. 1).

### Down regulation of miRNA biogenesis after AFB1 treatment

**Figure 3:**
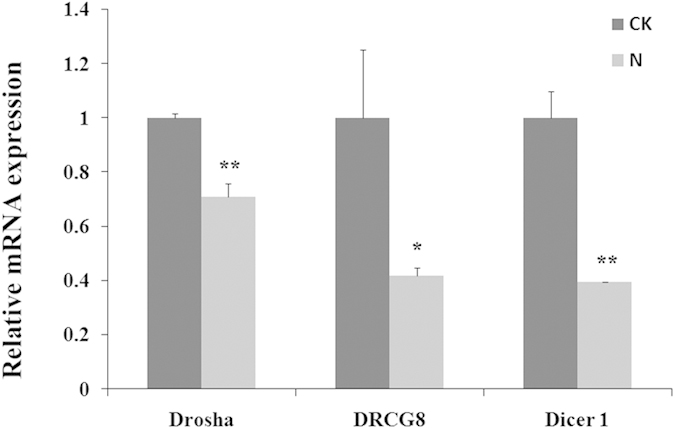
The mRNA expressions of *Drosha, DRCG8* and *Dicer1* of the HepG2 in CK and N group. HepG 2 cells were treated with AFB1 (0 and 10 μg/mL) for 24 hours, denoted as CK and N group. Expression levels were normalized using *β-actin*. Data are presented as the means ± SD. Three independent experiments are conducted in HepG2 cells. ^*^p < *0.05*, ^**^p < *0.01.*

The microprocessor component genes DiGeorge syndrome critical region 8 (*DGCR8*), *Drosha* and *Dicer* are the miRNA processing enzymes that are required for the maturation of miRNAs. They are reported to be down-regulated in the human cancer mostly, so we examined the expression of these three miRNA genes to estimate the influence of AFB1. *Dicer* and *DRCG8* displayed a robust 2.0-fold decrease in the HepG2 cell lines after AFB1 administration (*p* = *0.002* and *p* = *0.02* respectively). The *Drosha* displayed a 1.5-fold decrease in expression (*p* = *0*.*005*) (Fig. 3). These results indicated that AFB1 affected the mechanism of miRNAs synthesis processing.

### Differently expressed miRNAs

**Figure 4:**
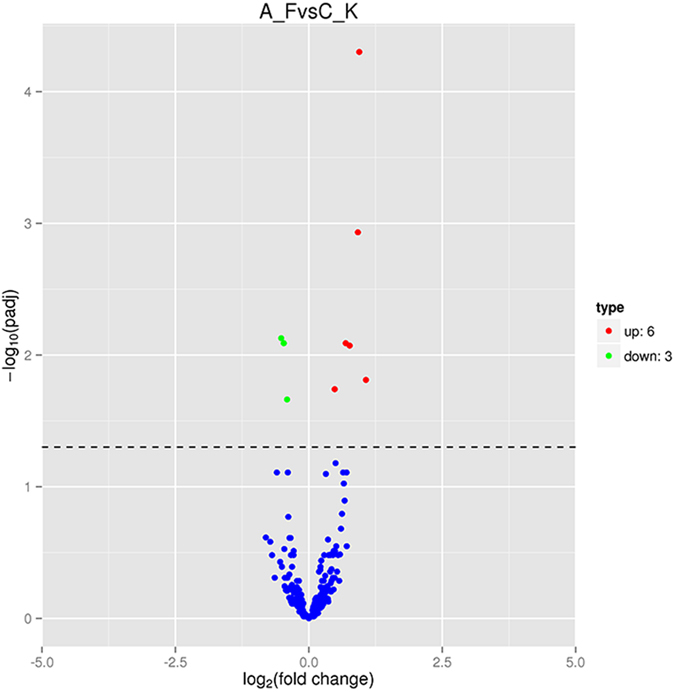
Different miRNA expression in volcano diagram. The x axis stands for the fold change of different miRNAs. The Y axis stands for significant difference of miRNA expression changes. Every miRNA are represented with the dots. The blue dots indicate no significant difference miRNAs; The red dots indicate up-regulated miRNAs; The green dots mean down-regulated miRNAs.

**Figure 5:**
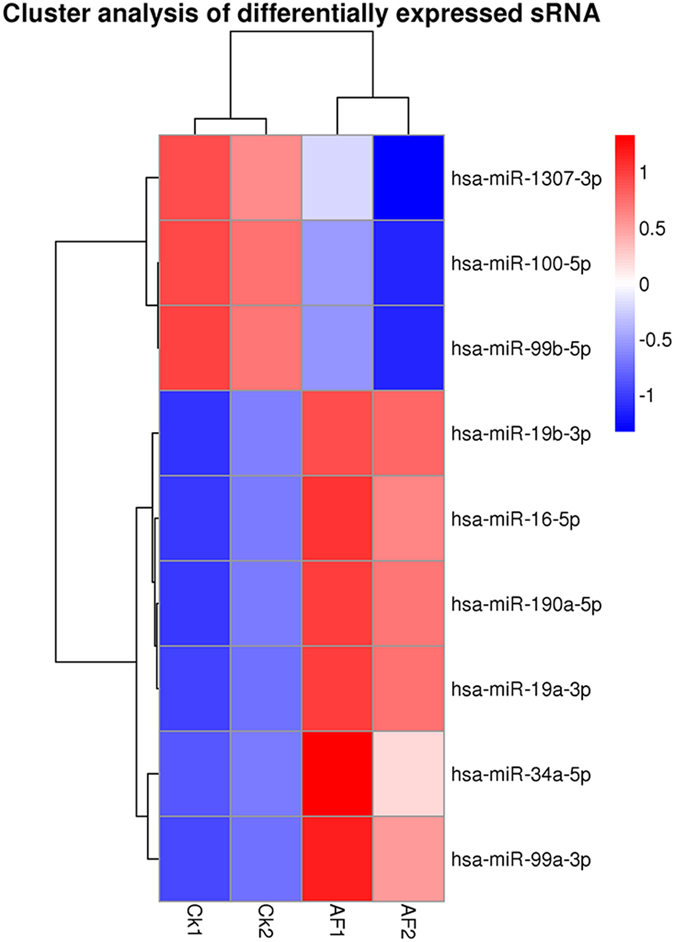
Hierarchical clustering of miRNA expression. miRNA profiles from four groups of HepG2 were clustered. Treatment groups are in columns, miRNAs in rows. Cluster analysis based on log10 (TPM + 1). The red means up-regulated miRNAs, and the blue means down-regulated miRNAs.

We further analyzed the differentially expressed miRNA between two conditions/groups using the DESeq2R package^28^. The read count data of the miRNA expression level was analyzed based on negative binomial distribution. Volcano Fig. 4 was used to show the global distribution of the differentially expressed miRNA, corrected P-value of 0.05 was set as the threshold for significantly differential expression by default (padj < 0.05) (Fig. 4).We used hierarchical cluster to analyze differentially expressed sRNA of every sample (Fig. 5). First we got a set of difference of miRNAs from each combination, then compared the TPM of every sample finite union from all the sets of differential expression miRNAs to make hierarchical cluster. The P-values was adjusted using the Benjamini & Hochberg method (padj < 0.05). The results showed that *hsa-miR-19b*, *hsa-miR-19a*, *hsa-miR-34a*, *hsa-miR-99a*, *hsa-miR-190a* and *hsa-miR-16* were up-regulated as a result of AFB1 treatment compared to CK, while *hsa-miR-1307*, *hsa-miR-99b*, *hsa-miR-100-5p* showed the opposite trend (Table 2).

**Figure 6:**
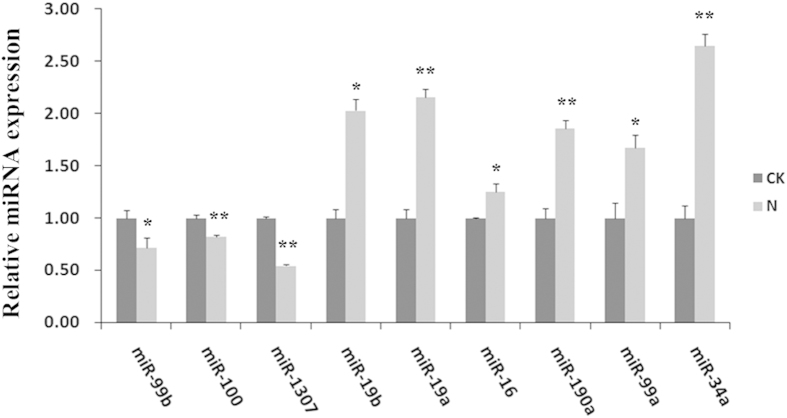
qRT-PCR analyses of 9 miRNAs expression levels. Expression levels were normalized by *5S RNA*. Data are presented as the means ± SD. Three independent experiments are conducted in HepG2 cells. ^*^p < *0.05*, ^**^p < *0.01*.

To validate the results, we analyzed the expression of these miRNA using qPCR, which revealed that *hsa-miR-19a* displayed a 2.15-fold increase in expression in the treatment group (p = 0.006), while *hsa-miR-1307* displayed a 0.54-fold decrease (p = 0.001) (Fig. 6). The detailed data were listed in the supplementary Table 2.

### The miRNA targets prediction and qRT-PCR validation

**Figure 7:**
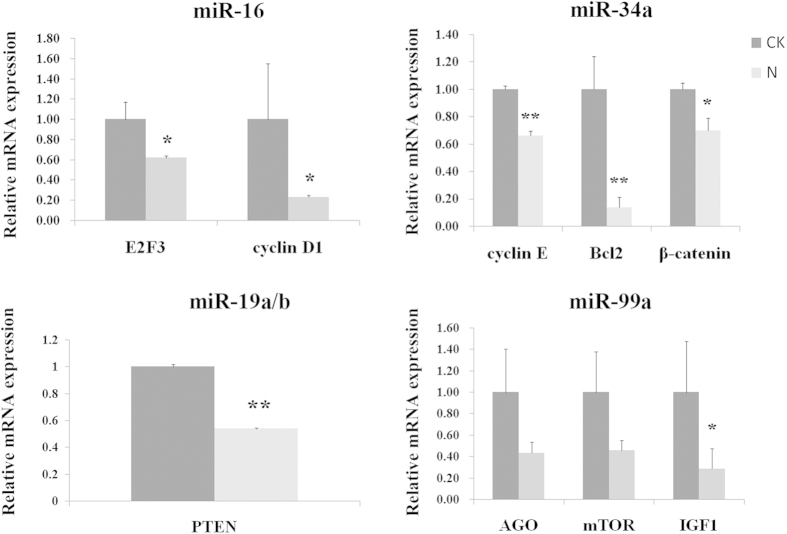
qRT-PCR analyses of mRNA expression levels. Expression levels were normalized using *β-actin*. Data are presented as the means ± SD. Three independent experiments are conducted in HepG2 cells. ^*^p < *0.05*, ^**^p < *0.01*.

The targets were predicted according to the method above by different miRNA target prediction algorithms, which can be helpful in minimizing the number of putative or false positive targets. The mRNA expression of *AGO/IGF1/mTOR* (*miR-99a*), *E2F3/Cyclin D1*(*miR-16*), *PTEN* (*miR-19a/b*), *Cyclin E/β-catenin/Bcl2* (*miR-34a*) were strongly correlated with its corresponding miRNAs shown in the parentheses. The mRNAs were decreased significantly after AFB1 treatment. All the primers used in the RT-PCR analyses were listed in supplementary Table 1B. The expression tendency of these mRNA targets was opposite to the expression of their corresponding miRNAs, as shown in Fig. 7.

### KEGG pathway and GO Analysis

The predicted target genes were subjected to KEGG pathway enrichment analysis (Supplementary Table 3). Six pathways including “Axon guidance” “Pathways in cancer” and “*Wnt* signaling pathway” were analyzed in both miRNA expression groups (all up-regulated miRNA were divided into one group while down-regulated ones the other). The *Wnt* signaling pathway has been studied to have a closely relationship with AFB1-induced hepatocellular cancers.

**Figure 8:**
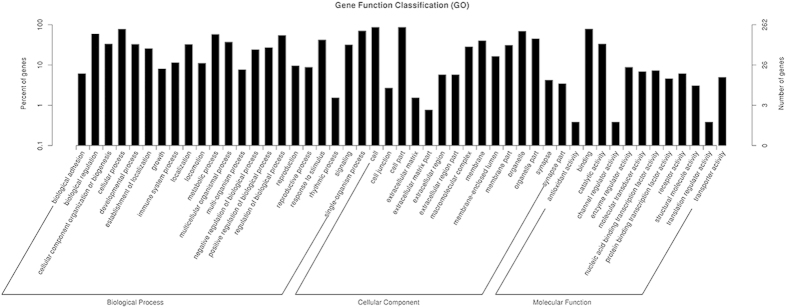
Function classification of the target genes of 9 miRNAs. GO terms were applied to enriched the target genes.

The top 30 enriched GO terms based on FDR for gene targets of the differently expressed miRNAs were shown in Table 3. Detailed classifications about the biological process, cellular component, and molecular function of the miRNA targets were shown in Fig. 8. Twenty one of the top 30 GO terms belonged to biological process, including “microtubule-basedprocess” “single-organism process” “biological regulation”. The other 9 GO terms were cellular component including “intracellular” “organelle”.

**Figure 9:**
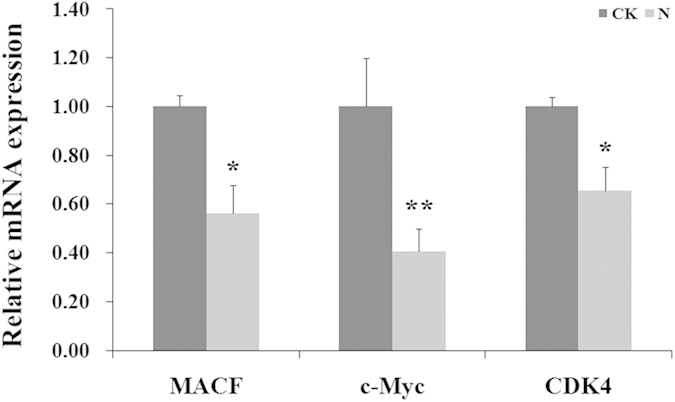
qRT-PCR analyses of the mRNA expression levels of *MACF1, c-Myc* and *CDK4*. Expression levels were normalized using *β-actin.* Data are presented as the means ± SD. Three independent experiments are conducted in HepG2 cells. ^*^p < *0.05*, ^**^p < *0.01*.

For further study on the regulatory mechanism of miRNA after AFB1 treatment, we focus on the *Wnt* signaling pathway, based on the results that several miRNA targets, such as *Cyclin D1*, all included in the *Wnt* signaling pathway. The other genes in this pathway, including *MACF1*, *CDK4*, and *c-Myc*, were all validated to have a decrease trend after AFB1 treatment (Fig. 9).

### Anti-miR-34a oligo transfection negatively correlates to levels of β-catenin and Wnt signaling pathway

**Figure 10:**
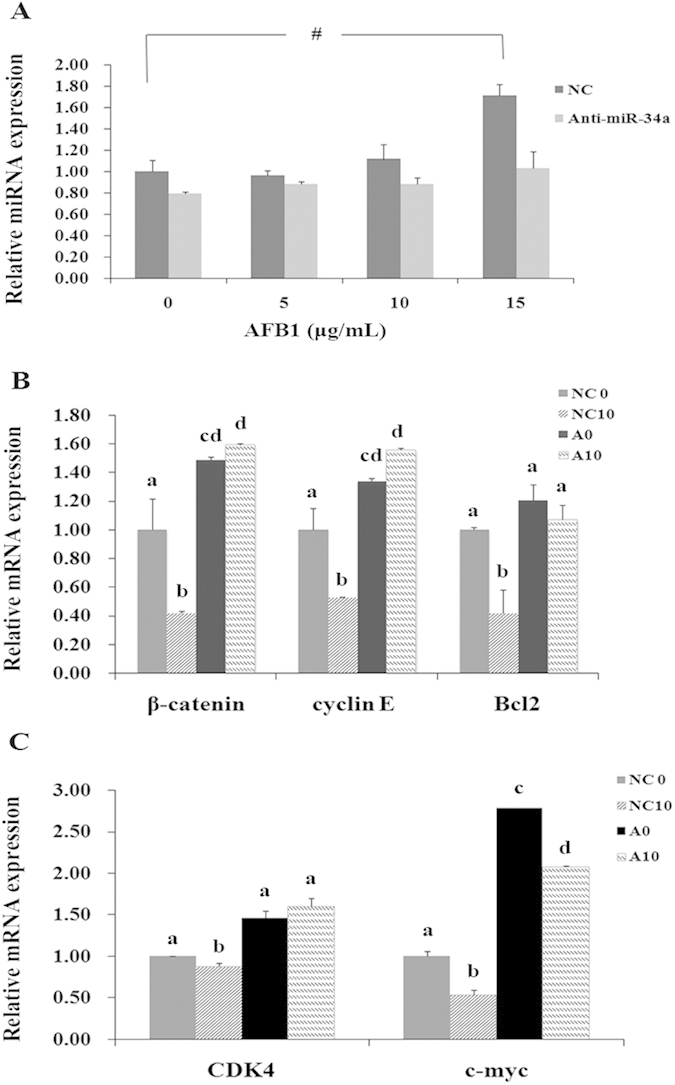
qRT-PCR analyses of miRNAs and genes expression. The detection was conducted in HepG2 cells transfected with an NC-inhibitor or *hsa-miR-34a* before treated with AFB1 (0, 5, 10 and 15 μg/mL). (**A**) The expression of *miR-34a* was detected. Expression levels were normalized using *5S.*^#^*p* < *0.05* (**B**) The mRNA expression levels of *β-catenin*, *cyclin E* and *Bcl-2*. Expression levels were normalized using *β-actin*. (**C**) The expression levels of *CDK4*, *c-myc* of the *Wnt* signaling pathway. Expression levels were normalized using *β-actin*. The significant difference were labelled with a, b, c and d. Data are presented as the means ± SD. Three independent experiments are conducted.

We previously showed that AFB1 down-regulated *β-catenin* expression and *Wnt* signaling pathway. Thus, to further test whether abnormal expression of *miR-34a* could induce its target genes to regulate cellular processes, we treated HepG2 cells with 150 nM *anti-miR-34a* oligo transfection before AFB1 adminstration, and performed qRT-PCR to detect. The results indicated that 150nM can effectively inhibited the *miR-34a* expression, and the levels of *miR-34a* were up-regulated in after AFB1 treatment both in NC group (marked NC) and *anti-miR-34a* group (marked A) (Fig. 10A). The *miR-34a* target gene, *β-catenin*, *cyclin E* and *Bcl2* (Fig. 10B), and *CDK4*, *c-Myc* of the *Wnt* signaling pathway all showed the opposite trend of *miR-34a* change (Fig. 10C). *Cyclin D1* showed no significant change.

### Deregulated miRNAs involved in the cell cycle

**Figure 11:**
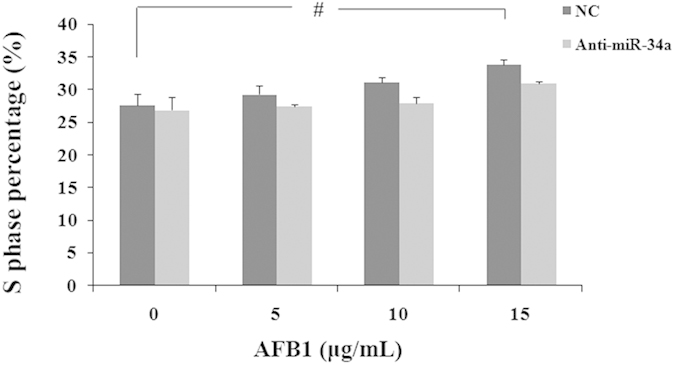
Cell-cycle analyses in NC and *anti-miR-34a* inhibitor group. The S phase of HepG2 cells transfected with an NC-inhibitor or *hsa-miR-34a* before treated with AFB1(0, 5, 10 and 15 μg/mL) were detected. Data are presented as the means ± SD. Three independent experiments are conducted. ^#^*p* < *0.05*.

The results analyzed by Modifit show that AFB1 induced S-phase arrest in HepG2 cell lines, especially the arrest were significant when 10 and 15 μg/mL AFB1 treatment (p = 0.053 and .003, respectively). However, the 150 nM *hsa-miR-34a* inhibitor seemed to reduce the S-phase arrest, which seemed there was no signification in the *hsa-miR-34a* inhibitor group. It can be seen in the Fig. 11, between the NC and *hsa-miR-34a* inhibitor group, the S-phase arrest were reduced significantly in 10 and 15 μg/mL AFB1 as shown. The G0/G1 phases were reversed with the S phase, while the G2/M phases were unchanged (Data were not shown).

## Discussion

Previous studies have revealed that AFB1 could induce cytotoxicity in hepatoma cells. Several studies also revealed that miRNAs aberrantly expressed in human HCC^29^. However, if miRNAs are involved in cell regulation of AFB1-induced HCC has received little attention. Previously, *Yang et al.* provided miRNA level changes in AFB1-induced hepatic injury which may lead to HCC through high-throughput profiling of miRNA in rat liver tissue before and after treatment with AFB1^30^. To further study the functional complexity of miRNAs in AFB1-induced HCC, we applied Illumina sequencing to investigate high-throughput profiling of miRNA in HepG2 treated with AFB1 *in vitro*. Different from the AFB1 miRNA profiling *in vivo*, we studied further about an important miRNA which seemed regulate several signaling pathways. To our knowledge, this is the first study to investigate the miRNA profiling under AFB1 stress and screen one single miRNA to further study its regulating pathways. These studies pave a new way to gain a better understanding of the mechanisms of AFB1 induced hepatocellular toxicology and carcinoma, in human cells.

The microprocessor component genes *DGCR8*, *Drosha* and the type III ribonuclease are responsible for cleavage of the pre-miRNA hairpin structure to form the mature miRNA. Both *Drosha* and *DGCR8* are abundant and ubiquitous, but the expression level of these proteins depends on cell types. *Dicer* has been revealed to degrade their target mRNAs which is required for the maturation of short interfering RNAs (siRNAs). Decreased *Dicer* expression in cancer conferred increased proliferative ability and an invasive phenotype^31^,^32^,^33^. Considering the important role of the integrity of miRNA processing mechanism, we studied *DGCR8*, *Drosha* and *Dicer*, which all displayed a decrease after 24 h AFB1 administration. Our expression analysis of the miRNA biosynthesis pathway revealed that three key molecules were simultaneously down-regulated in HepG2, indicating miRNA biosynthesis was damaged.

We generated a differential miRNA expression profile of HepG2 under different treatments (normal and AFB1 treated) and RT-PCR was performed to validate the profile. After background subtraction, normalization, and correction for multiple testing with SAM, we identified 9 miRNAs that were differentially expressed after AFB1 treatment. Significantly, 6 of these miRNAs were up-regulated, including several cancer-related miRNAs, *hsa-miR-34a*, *hsa-miR-19a/b* and *hsa-miR-99a*. However, these cancer-related miRNAs showed contradictory changes in expression relative to their known functions. For example, *miR-34a*, which is considered to be a tumour suppressive miRNA, was up-regulated in our study. In the past experiment, the over-expression of *miR-34a* was found in Fischer 344 male rats after AFB1 treatment for 3 days^30^, the parallel trend led us to the view that there may be different expression of miRNAs between the cancer development process and cancer formation.

We also predicted a set of potential target genes of the differentially expressed miRNAs. KEGG pathway and GO enrichment analysis based on the predicted targets revealed that the effect of abnormally expressed miRNAs upon HepG2 on a metabolomic scale. Based on the KEGG and GO analysis, we predicted the abnormally expressed miRNAs triggered by AFB1 might contribute to tumorigenesis in liver cancer, disorder of cell cycle and apoptosis. Furtherly, potential target genes of the differential expressed miRNAs, such as *AGO*/*IGF1*/*mTOR* (*miR-99a*), *E2F3*/*Cyclin D1* (*miR-16*), *PTEN* (*miR19a*/*b*), *Cyclin E*/*β-catenin*/*Bcl2* (*miR-34a*), were verified by q-PCR. *IGF-1R* and *mTOR* were characterized as direct targets of *miR-99a*, which exerted function of *miR-99a* as a cell cycle progression inhibitor. AGO is a key enzyme involved in the regulation of the processing step from pre-miRNA to mature miRNA, which is involve in HCC^34^. *PTEN* is a tumor suppressor gene and essential for regulating *PI3K*/*AKT* signaling pathway. There was a correlation of the down-regulation of *PTEN* mRNA with tumor TNM stage and metastasis in HCC^35^,^36^. We focus on the *miR-34a* and its targets in AFB1 administration. The previous studies showed that *Cyclin D1*, *cyclin E* and *β-catenin* are included in cell cycle induced by the *Wnt*/*β-catenin* signaling pathway, and *β-catenin* has been proved to be a critical component of this signaling pathway^37^. So we checked the expression of other key genes including *CDK4*, *MACF1* and *c-Myc,* and the results showed that these genes were all down-regulated by AFB1. The past experiments have studied that microtubule-actin cross-linking factor 1 (*MACF1*) appeared to be inactivate *GSK3β* by phosphorylation. Then, *β-catenin* was released and entered the nucleus. Our results showed the AFB1 exposure down-regulated *MACF1*, and then the expression of *β-catenin* with its downstream genes (*C-myc* and *cyclin D1*) was reduced as well^38^. *Cyclin D1*, with the help of *CDK4*, is a crucial mediator of the G1 to S progression^39^. The RT-PCR showed that *CDK4* and other member of the cyclin family, *Cyclin E*, were down-regulated upon exposure to AFB1. *C-Myc*, considered as the proto-oncogene, is always up-regulated in the tissue of cancer. However, it was down-regulated in AFB1-treated HepG2 cell. This indicated the different mechanisms of AFB1 *in vivo* and *in vitro*.

To further study, the *miR-34a* inhibitor was used before AFB1 treatment. RT-PCR showed that *miR-34a* inhibitor relieved the down expression of *miR-34a* targets genes and *CDK4*, *MACF1* and c-*myc*. Considering that these genes generally mediates cell cycle, we inspected the distribution of different phases of cell cycle. Cell cycle dysregulation was an essential step in the initiation and development of human malignancies, including HCC^40^. The results showed that AFB1 can induce S-phase arrest and the *miR-34a* inhibitor can relieved. We predicted that *miR-34a* induced *Cyclin D1* and *Cyclin E* down-regulated. there may be some other miRNAs or mechanisms induced S-phase arrest more than G1-arrest.

In summary, we preliminarily established the relationship between miRNAs, especially *miR-34a*, and AFB1 in HepG2. We applied Illumina sequencing and bioinformatics to compare the expression profile of AFB1-treated HepG2 and control HepG2 cell lines. We identified some abnormally expressed cancer-related miRNAs. This is the first study to investigate the miRNA expression and sequence profile in HepG2 under AFB1 administration. Among these abnormally expressed miRNA, *miR-34a* was proved to up-regulated by AFB1, which can participated in the regulation of *Wnt*/*β-catenin* signaling pathway and cell cycle. Based on multifaceted toxicology studies on AFB1, we hypothesized that AFB1 might trigger abnormal expression of miRNAs which are involve in the mechanism of liver tumorigenesis.

## Additional Information

**How to cite this article**: Zhu, L. *et al.* *miR-34a* screened by miRNA profiling negatively regulates *Wnt*/*β-catenin* signaling pathway in Aflatoxin B1 induced hepatotoxicity. *Sci. Rep.* **5**, 16732; doi: 10.1038/srep16732 (2015).

## Supplementary Material

Supplementary Information

## Figures and Tables

**Table 1:** Parameters of small RNA sequences.

Sample	Reads types	Reads member	Percentage
CK1	total_reads	11608636	100.00%
	N% > 10%	0	0.00%
	low quality	6805	0.06%
	5_adapter_contamine	2144	0.02%
	3_adapter_null or insert_null	222296	1.91%
	with polyA/T/G/C	24900	0.21%
	clean reads	11352491	97.79%
CK2	total_reads	11084457	100.00%
	N% > 10%	0	0.00%
	low quality	6055	0.06%
	5_adapter_contamine	2114	0.02%
	3_adapter_null or insert_null	212368	1.92%
	with polyA/T/G/C	23461	0.21%
	clean reads	10840459	97.80%
N1	total_reads	10482477	100.00%
	N% > 10%	0	0.00%
	low quality	5008	0.05%
	5_adapter_contamine	1997	0.02%
	3_adapter_null or insert_null	442269	4.22%
	with polyA/T/G/C	19394	0.19%
	clean reads	10013809	95.53%
N2	total_reads	14109860	100.00%
	N% > 10%	106	0.00%
	low quality	7233	0.05%
	5_adapter_contamine	2186	0.02%
	3_adapter_null or insert_null	303932	2.15%
	with polyA/T/G/C	33791	0.24%
	clean reads	13762612	97.54%

**Table 2:** Deferential expressed miRNAs between N and CK group.

	sRNA	N_readcount	CK_readcount	log 2 Fold Change	p-value	padj
**Up-regulated**	*hsa-miR-19b-3p*	1272.72	641.01	0.94	1.73E-07	5.00E-05
	*hsa-miR-19a-3p*	199.33	101.41	0.92	8.12E-06	1.17E-03
	*hsa-miR-34a-5p*	1215.53	734.77	0.69	7.76E-05	7.45E-03
	*hsa-miR-99a-3p*	182.44	103.67	0.77	1.41E-04	8.13E-03
	*hsa-miR-190a-5p*	61.72	25.97	1.07	1.15E-04	8.13E-03
	*hsa-miR-16-5p*	6711.27	4753.48	0.48	1.77E-04	8.47E-03
**Down-regulated**	*hsa-miR-1307-3p*	1518.55	2189.94	−0.52	3.76E-04	1.55E-02
	*hsa-miR-99b-5p*	3521.37	4909.88	−0.47	5.05E-04	1.82E-02
	*hsa-miR-100-5p*	64355.01	85846.86	−0.41	6.80E-04	2.18E-02

**Table 3:** The top 30 enriched GO terms.

	GO_accession	Description	Term_type	Corrected_pValue	CAD_item	Bg_item	Bg_list
1	GO:0005622	intracellular	cellular_component	0.082364	207	455	650
2	GO:0044424	intracellular part	cellular_component	0.082364	207	455	650
3	GO:0007017	microtubule-based process	biological_process	0.14989	20	25	650
4	GO:0044699	single-organism process	biological_process	0.14989	184	382	650
5	GO:0044763	single-organism cellular process	biological_process	0.14989	170	349	650
6	GO:0065007	biological regulation	biological_process	0.14989	154	312	650
7	GO:0032502	developmental process	biological_process	0.14989	86	155	650
8	GO:0048869	cellular developmental process	biological_process	0.14989	62	106	650
9	GO:0048856	anatomical structure development	biological_process	0.14989	77	135	650
10	GO:0005623	cell	cellular_component	0.14989	227	517	650
11	GO:0044464	cell part	cellular_component	0.14989	227	517	650
12	GO:0048731	system development	biological_process	0.16085	70	121	650
13	GO:0044707	single-multicellular organism process	biological_process	0.16085	94	174	650
14	GO:0032501	multicellular organismal process	biological_process	0.16232	97	182	650
15	GO:0042221	response to chemical stimulus	biological_process	0.16232	55	96	650
16	GO:0007275	multicellular organismal development	biological_process	0.16645	78	139	650
17	GO:0043226	organelle	cellular_component	0.19739	181	400	650
18	GO:0043227	membrane-bounded organelle	cellular_component	0.19739	162	332	650
19	GO:0043229	intracellular organelle	cellular_component	0.19739	180	398	650
20	GO:0030154	cell differentiation	biological_process	0.19739	58	100	650
21	GO:0010646	regulation of cell communication	biological_process	0.21052	46	76	650
22	GO:0065008	regulation of biological quality	biological_process	0.242	58	104	650
23	GO:0009966	regulation of signal transduction	biological_process	0.242	39	63	650
24	GO:0023051	regulation of signaling	biological_process	0.242	44	73	650
25	GO:0032940	secretion by cell	biological_process	0.25255	23	34	650
26	GO:0043231	intracellular membrane-bounded organelle	cellular_component	0.2665	160	330	650
27	GO:0048518	positive regulation of biological process	biological_process	0.2665	71	128	650
28	GO:0048583	regulation of response to stimulus	biological_process	0.27299	48	81	650
29	GO:0048513	organ development	biological_process	0.27299	53	92	650
30	GO:0005737	cytoplasm	cellular_component	0.28884	153	317	650
