# Supplementary material for: miR-34a screened by miRNA profiling negatively regulates Wnt/β-catenin signaling pathway in Aflatoxin B1 induced hepatotoxicity
Source: Sci Rep. 2015 Nov 16;5:16732. doi: 10.1038/srep16732 (PMC4645126; doi:10.1038/srep16732)
Supplement: Supplementary Information [file srep16732-s1.pdf]

miR-34a screened by miRNA profiling negatively regulates *Wnt/β-catenin* signaling pathway in Aflatoxin B1 induced hepatotoxicity

Liye Zhu[a], Jing Gao[a], Kunlun Huang[a, b], Yunbo Luo[a, b], Boyang Zhang[a],

Wentao Xu[a,b*]

[a]Laboratory of Food Safety and Molecular Biology, College of Food Science and Nutritional Engineering, China Agricultural University, Beijing, China, 100083

[b]Beijing Laboratory for Food Quality and Safety, Beijing, P. R. China

Running title: *miR-34a* negatively regulates *Wnt/β-catenin* pathway in AFB1 induced hepatotoxicity

[*]Corresponding author

Address: College of Food Science and Nutritional Engineering, China Agricultural University, No. 17 Tsinghua Donglu, Beijing, China, 100083.

Fax/Phone: (8610)62738793; E-mail: xuwentao@cau.edu.cn

Number of Figures: 11

Number of tables: 3

Table 1 Primers of miRNAs and genes.

A miRNAs primers of differential expression miRNAs and Novel miRNA.

Name	Sequence
19a-3p Forward	GGTGTGCAAATCTATGCAAAACTGA
19b-3p Forward	GTGTGCAAATCCATGCAAAACTGA
99a-3p Forward	CAAGCTCGCTTCTATGGGTCTG
190a-5p Forward	GGGGTGATATGTTTGATATATTAGGT
16-5p Forward	TAGCAGCACGTAAATATTGGCG
1307-3p Forward	ACTCGGCGTGGCGTCGGTCGTG
99b-5p Forward	CACCCGTAGAACCGACCTTGCG
100-5p Forward	AACCCGTAGATCCGAACTTGTG
34a-5p Forward	TGGCAGTGTCTTAGCTGGTTGT
novel_531 Forward	GGGCAAAGATGTTTCTCTTCCT
Uni Reverse	CTCAACTGGTGTCGTGGAGTC

B mRNAs primers of miRNA biogenesis genes, miRNA targets genes and Wnt signaling pathway genes

Name	Sequence
Drosha Forward	AGAGGCAATCAAGCCCTGTC
Drosha Reverse	GACTGTTGGCCTGTCCGTTA
Dicer Forward	GGAGAGTTACCCCAAACCCG
Dicer Reverse	TGTCCTCAGGAGGGTAGAGC
DGCR8 Forward	CTTGCTGCGCATGTATGGTC
DGCR8 Reverse	TCAGGATGTGAAGGTTGGGC
E2F3 Forward	CCAACTCAGGACATAGCGATTGCTC
E2F3 Reverse	AGGAATTTGGTCCTCAGTCTGCTGT
Cyclin-D1 Forward	CGCCCCACCCCTCCAG
Cyclin-D1 Reverse	CCGCCCAGACCCTCAGACT
AGO Forward	TCCACCTAGACCCGACTTT
AGO Reverse	GTTCCACGATTTCCCTGTT
mTOR Forward	ACA TGC AGC TGT CCT GGT TC
mTOR Reverse	TGA GGC TTC TGC ATC TCC TT
IGF-1R Forward	TGAAAGTGACGTCCTGCATTTC
IGF-1R Reverse	GGTACCGGTGCCAGGTTATG
PTEN Forward	TGGAAAGGGACGAACTGGTG
PTEN Reverse	CATAGCGCCTCTGACTGGGA
Cyclin-E Forward	GCAGTATCCCCAGCAAATC
Cyclin-E Reverse	TCAAGGCAGTCAACATCCA
BCL-2 Forward	ATGTGTGTGGAGAGCGTCAACC
BCL-2 Reverse	TGAGCAGAGTCTTCAGAGACAGCC
MET Forward	TGGTGCAGAGGAGCAATGG

MET Reverse	CATTCTGGATGGGTGTTTCCG
β-catenin Forward	GCTGGGACCTTGCATAACCTT
β-catenin Reverse	GCACAGAGGGCAACGAAGG
MACF1 Forward	CTCGGGCACTAATAGCACA
MACF1 Reverse	ACCATTTCCCCTTCCTCA
CDK4 Forward	CAGATGGCACTTACACCCGTG
CDK4 Reverse	GCAGCCCAATCAGGTCAAAGA
β-actin Forward	TCGTGCGTGACATTAAGGAG
β-actin Reverse	AGGAAGGAAGGCTGGAAGAG

Fig. 1 The detail information of the novel miRNA

>novel_5319:12455613..12455720 :- a minimum free energy of -27.40 kcal/mol

AUAAUGUCUGUGGCAUCGGGAAAGAUGUUUCUCUUCCUUUUAUUGGAAGUUGUAGAGA

GAUGUUUGCUAGUUUCUCAGAUUCUGUGGAUAAAGAUGAGUUGCCUAGAC

.....(((((.((((..((.(((.((.((((((((((.......)))))....))))).)).))).)).....((((...(((........))))))).)))))))))

Novel_531 TPM

sRNA.readcount	AF1.readcount	AF2.readcount	Ck1.readcount	Ck2.readcount
novel_531	5	14	6	9

Table 2 The differential analysis results of miRNAs.

sRNA	A_F_readcount	C_K_readcount	log2Fold Change	pval	padj	significant
hsa-let-7a-2-3p	9.249132127	11.46931317	-0.1511	0.71925	NA	NA
hsa-let-7a-3p	550.3607546	608.4382956	-0.14322	0.36	0.74589	FALSE
hsa-let-7a-5p	75712.47393	76305.2107	-0.010959	0.93269	0.96524	FALSE
hsa-let-7b-3p	94.61127939	98.91457313	-0.061696	0.81228	0.96524	FALSE
hsa-let-7b-5p	29396.12601	28012.29494	0.068101	0.5879	0.86828	FALSE
hsa-let-7c-3p	60.75772799	86.88056212	-0.44865	0.10078	0.49115	FALSE
hsa-let-7c-5p	10480.00616	9979.207152	0.069367	0.57067	0.8614	FALSE
hsa-let-7d-3p	567.5338447	570.7830711	-0.010934	0.94513	0.96524	FALSE
hsa-let-7d-5p	2744.50214	2811.565074	-0.033741	0.78237	0.96524	FALSE
hsa-let-7e-3p	29.32135325	26.7672915	0.064259	0.8733	NA	NA
hsa-let-7e-5p	2771.367689	2541.581909	0.11925	0.52604	0.83179	FALSE
hsa-let-7f-1-3p	16.0219773	15.10240979	0.064008	0.87051	NA	NA
hsa-let-7f-2-3p	82.90474098	66.31750351	0.30103	0.24084	0.69571	FALSE
hsa-let-7f-5p	117346.594	120040.4054	-0.029358	0.91028	0.96524	FALSE
hsa-let-7g-3p	2.363804228	2.258085391	-0.0093531	0.98043	NA	NA
hsa-let-7g-5p	44398.7271	48942.16468	-0.12912	0.58107	0.8671	FALSE
hsa-let-7i-3p	151.6611484	105.2416138	0.45465	0.099452	0.49115	FALSE
hsa-let-7i-5p	58917.16101	63448.67611	-0.097168	0.69317	0.93771	FALSE
hsa-miR-1	1.788247822	5.069511743	-0.50578	0.21057	NA	NA
hsa-miR-100-3p	6.577434881	3.454210415	0.39935	0.34985	NA	NA
hsa-miR-100-5p	64355.00797	85846.85944	-0.40739	0.00068019	0.021766	TRUE
hsa-miR-101-3p	6570.063204	5602.90003	0.22253	0.14457	0.57785	FALSE
hsa-miR-101-5p	0	1.151403472	-0.29275	0.2327	NA	NA
hsa-miR-103a-2-5p	3.381085346	1.704744432	0.30477	0.4402	NA	NA
hsa-miR-103a-3p	10983.42414	10321.43025	0.087727	0.5059	0.83179	FALSE
hsa-miR-103b	10842.53765	10156.76287	0.09224	0.47995	0.81312	FALSE
hsa-miR-105-3p	10.98647583	5.757017359	0.46741	0.27426	NA	NA
hsa-miR-105-5p	103.1830469	124.2621318	-0.25752	0.30154	0.72093	FALSE
hsa-miR-106a-5p	27.15295932	16.16437016	0.44054	0.28992	NA	NA
hsa-miR-106b-3p	2874.810995	3047.244307	-0.078615	0.68668	0.93771	FALSE
hsa-miR-106b-5p	3638.212279	2714.544077	0.39917	0.038806	0.32871	FALSE
hsa-miR-107	1735.455884	1407.612399	0.28342	0.15429	0.58466	FALSE
hsa-miR-10a-3p	498.8841822	476.0662925	0.060416	0.75895	0.96524	FALSE
hsa-miR-10a-5p	50533.68966	57184.23976	-0.16764	0.41006	0.77231	FALSE
hsa-miR-10b-5p	39.2585245	48.49311047	-0.26476	0.3684	0.74625	FALSE
hsa-miR-1179	0.63713501	0.55334096	0.02459	0.91475	NA	NA
hsa-miR-1180-3p	1392.021168	1335.10202	0.055613	0.76097	0.96524	FALSE

hsa-miR-1180-5p	0.575556406	0	0.10102	0.52785	NA	NA
hsa-miR-122-3p	0.318567505	0	0.069707	0.60336	NA	NA
hsa-miR-1224-5p	0.318567505	0	0.069707	0.60336	NA	NA
hsa-miR-122-5p	2.425382831	5.024790191	-0.32669	0.41113	NA	NA
hsa-miR-1226-3p	3.000939237	0.55334096	0.45748	0.20362	NA	NA
hsa-miR-1226-5p	0.63713501	0	0.12351	0.48089	NA	NA
hsa-miR-1228-3p	1.212691416	0	0.28818	0.26494	NA	NA
hsa-miR-1228-5p	0.318567505	0	0.069707	0.60336	NA	NA
hsa-miR-1229-3p	0.955702515	0.598062512	0.071279	0.77113	NA	NA
hsa-miR-1234-3p	0.894123911	0.55334096	0.067286	0.80504	NA	NA
hsa-miR-1246	72.5365195	65.45111168	0.11256	0.6895	0.93771	FALSE
hsa-miR-1247-3p	32.49635381	33.16709292	-0.039978	0.90248	0.96524	FALSE
hsa-miR-1247-5p	27.14228483	32.41818342	-0.21754	0.55344	NA	NA
hsa-miR-1248	4.984597356	7.506483342	-0.29935	0.48162	NA	NA
hsa-miR-1250-5p	97.45703797	92.35256784	0.079249	0.73648	0.95975	FALSE
hsa-miR-1254	25.64058105	35.78295073	-0.31491	0.41549	0.77231	FALSE
hsa-miR-1255a	31.51930232	32.19457566	0.00063182	0.99853	0.99853	FALSE
hsa-miR-1255b-5p	4.090473446	2.258085391	0.20597	0.59239	NA	NA
hsa-miR-1257	5.806468177	4.052272927	0.25491	0.55073	NA	NA
hsa-miR-125a-3p	18.76592764	26.67784839	-0.37241	0.32868	NA	NA
hsa-miR-125a-5p	4342.510122	5082.097944	-0.21825	0.19754	0.63212	FALSE
hsa-miR-125b-1-3p	104.508221	117.2195464	-0.16365	0.46036	0.80843	FALSE
hsa-miR-125b-2-3p	970.8625206	1138.201375	-0.22118	0.11636	0.5183	FALSE
hsa-miR-125b-5p	7089.930338	7528.067937	-0.082666	0.65355	0.92266	FALSE
hsa-miR-1260a	0.955702515	3.364767311	-0.3364	0.33555	NA	NA
hsa-miR-1260b	259.7860947	231.6890703	0.1475	0.47997	0.81312	FALSE
hsa-miR-1262	8.365682702	5.293119503	0.29776	0.48647	NA	NA
hsa-miR-126-3p	413.2305542	408.9491265	0.014683	0.9343	0.96524	FALSE
hsa-miR-126-5p	42.96885184	28.51675748	0.46387	0.16542	0.60305	FALSE
hsa-miR-1266-5p	20.02952317	17.69022838	0.14451	0.72012	NA	NA
hsa-miR-1268a	132.1299919	177.1425739	-0.3396	0.30392	0.72093	FALSE
hsa-miR-1271-5p	0.575556406	1.794187536	-0.16374	0.5265	NA	NA
hsa-miR-1272	0.63713501	0	0.12351	0.48089	NA	NA
hsa-miR-1273c	3.44266395	4.560892335	-0.11212	0.78517	NA	NA
hsa-miR-1273d	1.469680317	1.704744432	-0.072687	0.83594	NA	NA
hsa-miR-1273e	0	0.598062512	-0.12115	0.46355	NA	NA
hsa-miR-1273g-3p	1.274270019	1.151403472	0.044559	0.88526	NA	NA
hsa-miR-1273h-3p	0.63713501	0	0.12351	0.48089	NA	NA
hsa-miR-1273h-5p	0.318567505	1.196125024	-0.14255	0.55688	NA	NA

hsa-miR-1275	1.592837524	0.55334096	0.15591	0.55544	NA	NA
hsa-miR-1276	3.956641752	2.811426351	0.17472	0.67002	NA	NA
hsa-miR-1277-3p	6.197288772	1.749465984	0.57	0.16271	NA	NA
hsa-miR-1277-5p	151.3023512	106.4377388	0.41536	0.1892	0.62631	FALSE
hsa-miR-1278	73.95282738	111.3730859	-0.50378	0.067061	0.40536	FALSE
hsa-miR-128-1-5p	15.69273531	22.60889313	-0.32705	0.41208	NA	NA
hsa-miR-1283	2.106815326	1.749465984	0.067545	0.85754	NA	NA
hsa-miR-128-3p	3154.711272	3507.365218	-0.14799	0.35388	0.74481	FALSE
hsa-miR-1284	0.894123911	2.945591007	-0.37775	0.29255	NA	NA
hsa-miR-1285-3p	79.85289762	68.83256133	0.17922	0.55817	0.85054	FALSE
hsa-miR-1285-5p	9.629278235	7.880938094	0.1456	0.73258	NA	NA
hsa-miR-1286	3.319506742	1.749465984	0.2804	0.48007	NA	NA
hsa-miR-1287-3p	2.106815326	0.598062512	0.30492	0.36247	NA	NA
hsa-miR-1287-5p	41.29308674	41.31636033	0.0095153	0.97446	0.98128	FALSE
hsa-miR-1288-3p	1.151112812	0	0.16702	0.40499	NA	NA
hsa-miR-1291	12.00375695	5.248397951	0.65113	0.12596	NA	NA
hsa-miR-1292-5p	7.974862107	6.399801423	0.1916	0.65469	NA	NA
hsa-miR-1293	1.274270019	4.052272927	-0.39622	0.29966	NA	NA
hsa-miR-1294	5.292490375	2.302806944	0.42985	0.29318	NA	NA
hsa-miR-1295a	0.318567505	1.749465984	-0.30144	0.32367	NA	NA
hsa-miR-1295b-5p	0	1.749465984	-0.40511	0.14982	NA	NA
hsa-miR-129-5p	3.000939237	1.151403472	0.34705	0.36359	NA	NA
hsa-miR-1296-5p	188.165048	160.0450826	0.20582	0.37934	0.75345	FALSE

Table 3 KEGG enrichment analysis

Pathways	Each pathway targeted by different miRNAs	hsa-miR-100-5p	hsa-miR-1307-3p	hsa-miR-99b-5p
hsa04360 Axon guidance	3	1	1	1
hsa05200 Pathways in cancer	3	1	1	1
hsa04370 VEGF signaling pathway	3	1	1	1
hsa04914 Progesterone-mediated oocyte maturation	3	1	1	1
hsa04662 B cell receptor signaling pathway	3	1	1	1
hsa05223 Non-small cell lung cancer	3	1	1	1
hsa05222 Small cell lung cancer	3	1	1	1
hsa04722 Neurotrophin signaling pathway	3	1	1	1
hsa05221 Acute myeloid leukemia	3	1	1	1
hsa04670 Leukocyte transendothelial migration	3	1	1	1
hsa05220 Chronic myeloid leukemia	3	1	1	1
hsa04960 Aldosterone-regulated sodium reabsorption	3	1	1	1
hsa04310 Wnt signaling pathway	3	1	1	1
hsa05214 Glioma	3	1	1	1
hsa05217 Basal cell carcinoma	3	1	1	1
hsa04910 Insulin signaling pathway	3	1	1	1
hsa04720 Long-term potentiation	3	1	1	1
hsa04010 MAPK signaling pathway	3	1	1	1
hsa04150 mTOR signaling pathway	3	1	1	1
hsa04210 Apoptosis	2	1	1	0
hsa04012 ErbB signaling pathway	2	0	1	1
hsa04920 Adipocytokine signaling pathway	2	0	1	1
hsa04930 Type II diabetes mellitus	2	0	1	1
hsa04144 Endocytosis	2	0	1	1
hsa05212 Pancreatic cancer	2	0	1	1
hsa00604 Glycosphingolipid biosynthesis - ganglio series	2	1	0	1

hsa05211 Renal cell carcinoma	2	0	1	1
hsa04664 Fc epsilon RI signaling pathway	2	0	1	1
hsa04020 Calcium signaling pathway	2	0	1	1
hsa04510 Focal adhesion	2	0	1	1
hsa04070 Phosphatidylinositol signaling system	2	1	1	0
hsa05210 Colorectal cancer	2	0	1	1
hsa05142 Chagas disease	2	0	1	1
hsa05218 Melanoma	2	0	1	1
hsa04666 Fc gamma R-mediated phagocytosis	1	0	1	0
hsa00450 Selenoamino acid metabolism	1	0	1	0
hsa04270 Vascular smooth muscle contraction	1	0	1	0
hsa05014 Amyotrophic lateral sclerosis (ALS)	1	0	1	0
hsa00532 Glycosaminoglycan biosynthesis - chondroitin sulfate	1	0	1	0
hsa04520 Adherens junction	1	0	1	0
hsa04330 Notch signaling pathway	1	0	1	0
hsa04514 Cell adhesion molecules (CAMs)	1	0	1	0
hsa04142 Lysosome	1	0	1	0
hsa04120 Ubiquitin mediated proteolysis	1	0	0	1
hsa04114 Oocyte meiosis	1	0	1	0
hsa05216 Thyroid cancer	1	0	1	0
hsa05120 Epithelial cell signaling in Helicobacter pylori infection	1	0	1	0
hsa04962 Vasopressin-regulated water reabsorption	1	0	1	0
hsa05410 Hypertrophic cardiomyopathy (HCM)	1	0	1	0
hsa05414 Dilated cardiomyopathy	1	0	1	0
hsa04660 T cell receptor signaling pathway	1	0	1	0
hsa04971 Gastric acid secretion	1	0	1	0
hsa05215 Prostate cancer	1	0	1	0
hsa04540 Gap junction	1	0	1	0
hsa05131 Shigellosis	1	0	1	0
hsa04970 Salivary secretion	1	1	0	0
hsa04062 Chemokine signaling pathway	1	0	1	0
hsa05213 Endometrial cancer	1	0	1	0
hsa04530 Tight junction	1	1	0	0
hsa04810 Regulation of actin cytoskeleton	1	0	1	0
hsa04912 GnRH signaling pathway	1	0	1	0
hsa04916 Melanogenesis	1	0	1	0
hsa04730 Long-term depression	1	0	1	0
hsa00564 Glycerophospholipid metabolism	1	0	1	0

Pathways	Each pathway targeted by different miRNAs	hsa-miR-16-5p	hsa-miR-190a-5p	hsa-miR-19a-3p	hsa-miR-19b-3p	hsa-miR-34a-5p	hsa-miR-99a-3p
hsa04360 Axon guidance	6	1	1	1	1	1	1
hsa05200 Pathways in cancer	6	1	1	1	1	1	1
hsa04210 Apoptosis	6	1	1	1	1	1	1
hsa04012 ErbB signaling pathway	6	1	1	1	1	1	1
hsa05223 Non-small cell lung cancer	6	1	1	1	1	1	1
hsa04722 Neurotrophin signaling pathway	6	1	1	1	1	1	1
hsa04520 Adherens junction	6	1	1	1	1	1	1
hsa04920 Adipocytokine signaling pathway	6	1	1	1	1	1	1
hsa05221 Acute myeloid leukemia	6	1	1	1	1	1	1
hsa05212 Pancreatic cancer	6	1	1	1	1	1	1
hsa05220 Chronic myeloid leukemia	6	1	1	1	1	1	1
hsa04120 Ubiquitin mediated proteolysis	6	1	1	1	1	1	1
hsa05211 Renal cell carcinoma	6	1	1	1	1	1	1
hsa04310 Wnt signaling pathway	6	1	1	1	1	1	1
hsa04660 T cell receptor signaling pathway	6	1	1	1	1	1	1
hsa04971 Gastric acid secretion	6	1	1	1	1	1	1
hsa05214 Glioma	6	1	1	1	1	1	1
hsa05215 Prostate cancer	6	1	1	1	1	1	1
hsa04020 Calcium signaling pathway	6	1	1	1	1	1	1
hsa04910 Insulin signaling pathway	6	1	1	1	1	1	1
hsa04510 Focal adhesion	6	1	1	1	1	1	1
hsa04070 Phosphatidylinositol signaling system	6	1	1	1	1	1	1
hsa04720 Long-term potentiation	6	1	1	1	1	1	1
hsa04010 MAPK signaling pathway	6	1	1	1	1	1	1
hsa04150 mTOR signaling pathway	6	1	1	1	1	1	1
hsa05213 Endometrial cancer	6	1	1	1	1	1	1
hsa05218 Melanoma	6	1	1	1	1	1	1

hsa04370 VEGF signaling pathway	5	1	0	1	1	1	1
hsa04914 Progesterone-mediated oocyte maturation	5	1	1	1	1	1	0
hsa04662 B cell receptor signaling pathway	5	1	1	1	1	1	0
hsa05222 Small cell lung cancer	5	1	1	1	1	1	0
hsa04930 Type II diabetes mellitus	5	1	1	1	1	1	0
hsa04144 Endocytosis	5	1	0	1	1	1	1
hsa04340 Hedgehog signaling pathway	5	1	0	1	1	1	1
hsa04540 Gap junction	5	1	1	1	1	1	0
hsa05210 Colorectal cancer	5	1	1	1	1	1	0
hsa04062 Chemokine signaling pathway	5	1	0	1	1	1	1
hsa05142 Chagas disease	5	1	1	1	1	1	0
hsa04810 Regulation of actin cytoskeleton	5	1	1	1	1	1	0
hsa04912 GnRH signaling pathway	5	1	0	1	1	1	1
hsa04916 Melanogenesis	5	1	0	1	1	1	1
hsa04730 Long-term depression	5	1	0	1	1	1	1
hsa04666 Fc gamma R-mediated phagocytosis	4	1	0	1	1	1	0
hsa04270 Vascular smooth muscle contraction	4	1	0	0	1	1	1
hsa00562 Inositol phosphate metabolism	4	1	1	0	1	1	0
hsa05219 Bladder cancer	4	1	0	1	1	1	0
hsa04710 Circadian rhythm - mammal	4	1	0	1	1	1	0
hsa04114 Oocyte meiosis	4	1	0	1	1	1	0
hsa05216 Thyroid cancer	4	1	0	1	1	1	0
hsa04960 Aldosterone-regulated sodium reabsorption	4	1	1	0	1	1	0
hsa04350 TGF-beta signaling pathway	4	1	0	1	1	1	0
hsa05217 Basal cell carcinoma	4	1	0	1	1	1	0
hsa04130 SNARE interactions in vesicular transport	4	1	0	1	1	1	0
hsa05100 Bacterial invasion of epithelial cells	4	1	0	1	1	1	0
hsa04141 Protein processing in endoplasmic reticulum	4	0	0	1	1	1	1
hsa04115 p53 signaling pathway	4	1	1	0	1	1	0
hsa05014 Amyotrophic lateral sclerosis (ALS)	3	0	1	1	0	1	0
hsa04514 Cell adhesion molecules (CAMs)	3	1	0	0	1	1	0
hsa04670 Leukocyte transendothelial migration	3	0	0	1	1	1	0
hsa04142 Lysosome	3	1	0	0	0	1	1
hsa05120 Epithelial cell signaling in Helicobacter pylori infection	3	0	0	1	1	1	0
hsa05410 Hypertrophic cardiomyopathy (HCM)	3	0	0	1	1	1	0
hsa05414 Dilated cardiomyopathy	3	0	0	1	1	1	0
hsa04664 Fc epsilon RI signaling pathway	3	1	0	0	1	1	0
hsa05131 Shigellosis	3	1	0	1	0	1	0
hsa05412 Arrhythmogenic right ventricular cardiomyopathy (ARVC)	3	0	0	1	1	1	0
hsa04530 Tight junction	3	0	0	1	1	1	0
hsa00532 Glycosaminoglycan biosynthesis - chondroitin sulfate	2	1	0	0	0	1	0
hsa04512 ECM-receptor interaction	2	0	1	0	0	1	0

hsa04080 Neuroactive ligand-receptor interaction	2	0	0	1	0	1	0
hsa05146 Amoebiasis	2	0	1	0	0	1	0
hsa00534 Glycosaminoglycan biosynthesis - heparan sulfate	2	1	0	0	0	1	0
hsa04970 Salivary secretion	2	1	0	0	0	1	0
hsa00564 Glycerophospholipid metabolism	2	1	0	0	0	1	0
hsa04621 NOD-like receptor signaling pathway	1	0	0	0	0	1	0
hsa00030 Pentose phosphate pathway	1	1	0	0	0	0	0
hsa04330 Notch signaling pathway	1	0	0	0	0	1	0
hsa00071 Fatty acid metabolism	1	0	0	0	0	1	0
hsa00410 beta-Alanine metabolism	1	0	0	0	0	1	0
hsa00770 Pantothenate and CoA biosynthesis	1	0	0	0	0	1	0
hsa01100 Metabolic pathways	1	0	0	0	0	1	0
hsa00601 Glycosphingolipid biosynthesis - lacto and neolacto series	1	0	0	0	0	1	0
hsa04620 Toll-like receptor signaling pathway	1	0	0	0	0	1	0
hsa04630 Jak-STAT signaling pathway	1	0	0	0	0	1	0
hsa00280 Valine, leucine and isoleucine degradation	1	0	0	0	0	1	0
hsa04962 Vasopressin-regulated water reabsorption	1	0	0	0	0	1	0
hsa05110 Vibrio cholerae infection	1	0	0	0	1	0	0
hsa00561 Glycerolipid metabolism	1	1	0	0	0	0	0
hsa00533 Glycosaminoglycan biosynthesis - keratan sulfate	1	0	0	0	0	1	0
hsa03320 PPAR signaling pathway	1	0	0	0	0	1	0
hsa04146 Peroxisome	1	0	0	0	0	1	0
hsa00062 Fatty acid elongation in mitochondria	1	0	0	0	0	1	0
hsa05020 Prion diseases	1	0	0	0	0	1	0
hsa00520 Amino sugar and nucleotide sugar metabolism	1	0	0	0	0	1	0
hsa04110 Cell cycle	1	0	0	0	0	1	0
hsa00760 Nicotinate and nicotinamide metabolism	1	0	0	0	0	1	0
hsa00640 Propanoate metabolism	1	0	0	0	0	1	0
hsa04320 Dorso-ventral axis formation	1	0	0	0	0	1	0
hsa02010 ABC transporters	1	0	0	0	0	1	0
